# Re-framing bio-plausible collision detection: identifying shared meta-properties through strategic prototyping

**DOI:** 10.3389/fnbot.2024.1349498

**Published:** 2024-01-25

**Authors:** Haotian Wu, Shigang Yue, Cheng Hu

**Affiliations:** ^1^School of Mechanical and Electrical Engineering, Guangzhou University, Guangzhou, China; ^2^Machine Life and Intelligence Research Center, Guangzhou University, Guangzhou, China; ^3^School of Computing and Mathematical Sciences, University of Leicester, Leicester, United Kingdom

**Keywords:** bio-plausible, motion detection, visual sensing, robot, environment perception, LGMD

## Abstract

Insects exhibit remarkable abilities in navigating complex natural environments, whether it be evading predators, capturing prey, or seeking out con-specifics, all of which rely on their compact yet reliable neural systems. We explore the field of bio-inspired robotic vision systems, focusing on the locust inspired Lobula Giant Movement Detector (LGMD) models. The existing LGMD models are thoroughly evaluated, identifying their common meta-properties that are essential for their functionality. This article reveals a common framework, characterized by layered structures and computational strategies, which is crucial for enhancing the capability of bio-inspired models for diverse applications. The result of this analysis is the Strategic Prototype, which embodies the identified meta-properties. It represents a modular and more flexible method for developing more responsive and adaptable robotic visual systems. The perspective highlights the potential of the Strategic Prototype: LGMD-Universally Prototype (LGMD-UP), the key to re-framing LGMD models and advancing our understanding and implementation of bio-inspired visual systems in robotics. It might open up more flexible and adaptable avenues for research and practical applications.

## 1 Introduction

In the realm of robotics, the ability to detect impending collisions is essential for navigation and interaction with dynamic environments. Conventional methods employed for visual detection in robotics are often hard-coded and rigid, typically require large amounts of data for training, lacking the necessary adaptability to respond to the complex and sophisticated movements encountered in real-world settings.

The natural world, particularly the realm of insects, offers valuable lessons on visual processing under complex scenes. Like robots, many insect species face similar visual challenges to navigate and survive in a high dynamic environment (Borst et al., 2010; Borst and Helmstaedter, 2015). These challenges are visual motion perceptions that the animals perceive and calculate through their highly effective visual neural structures. They display an exceptional capacity to detect dynamic motion visually, a key skill for avoiding predators and navigating through complex environments. Neuro-physiological and anatomical studies indicate that these abilities are dependent on designated sensory neural pathways (Judge and Rind, 1997; Peron and Gabbiani, 2009; Zhu et al., 2018). Researchers have already taken note of these abilities and used it as inspiration to develop new, biologically plausible neural models in the field of robotics, e.g., Meng et al. (2010) and Hu et al. (2017).

Within this context, the Locusts' Lobula Giant Movement Detectors (LGMDs) and its related neural models have garnered considerable interest. These models have been comprehensively analyzed, researched, and replicated for their proficiency in detecting swift motion cues of looming, which indicates the presence of a nearby object (Fu et al., 2019; Chang et al., 2023). Despite significant advances, these models still face challenges in flexibility and adaptability in our view.

Our perspective contributes to this rapidly-growing field by uncovering a compelling pattern: across different models and applications, we found a common framework, characterized by simple yet effective computational strategies. By examining the meta-properties of the LGMD models, we suggest that a wider outlook permits the combination of these models into a single paradigm. The renewed approach may be the key to re-framing LGMD models, thus advancing our understanding and implementation of bio-inspired visual systems, simplifying the route to generalization. It presents an innovative approach, offering a more adaptable framework over LGMD models, marking a significant advancement in the methodology of robotic vision systems.

Manipulating meta-properties offers great potential for innovation, enabling the exploration of new directions in research and practical applications. It has the potential to enhance robotic visual systems, as well as catalyze the emergence of new ideas, ushering in an era of more intuitive and adaptive robotic perception. These advancements may prove promising in the realms of micro-robot navigation, automatic driving, and swarm robotics.

## 2 LGMD insights

**Figure 1:**
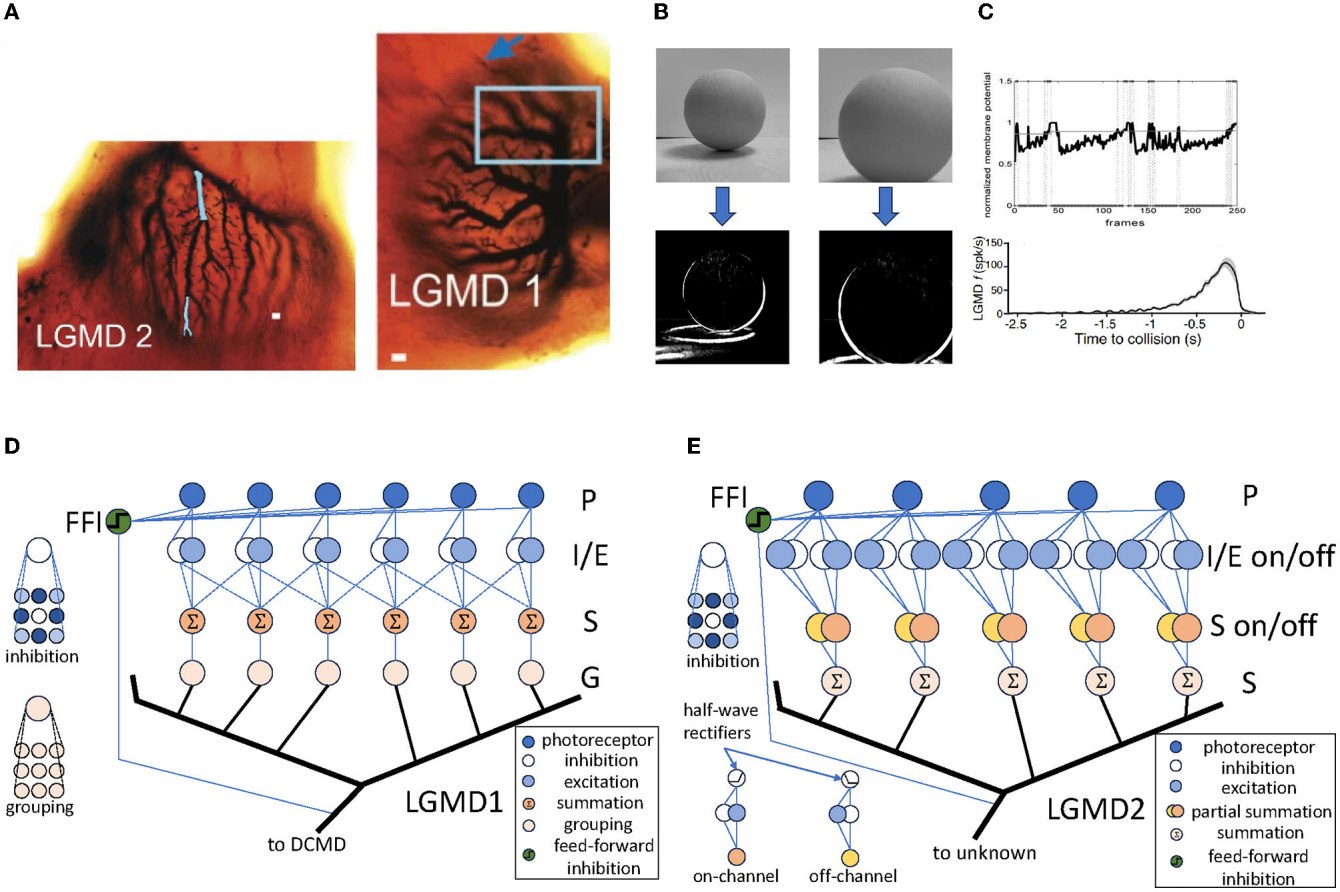
The LGMD neurons and their computational models. **(A)** The structure of LGMD1 and LGMD2 neurons in locust, which is adapted from Rind et al. (2016). The figure depicts the abundant dendrite trees with fan-shaped structures in the pre-synaptic regions of both neurons. The scale bars in LGMD1 and LGMD2 are 10 and 5μ*m*, respectively. **(B)** The responds to a computer-generated visual stimuli of an LGMD1 model, illustrating the middle layer's response of the model, which effectively identified the shape of the looming object. The model is based on Yue and Rind (2006). **(C)** The responds of the LGMD models and neurons, which is adapted from Dewell and Gabbiani (2018). The standard LGMD neuron response is depicted in the upper, whilst the lower portrays the LGMD model's response identically to the actual neuron. **(D)** One possible LGMD1 model, which is based on Yue and Rind (2006). This is a standard LGMD1 model with four feed-forward connection layers and one hyper-layer connection layer. **(E)** One possible LGMD2 model, which is based on Fu and Yue (2015). This is a typical LGMD2 model, similar to LGMD1, with “on” and “off” pathways split from the I/E layer and then reconnected in the S layer.

The LGMD neurons are first discovered in locust by O'Shea and Williams (1974), and has been tested, As illustrated in Figure 1A, the LGMD neurons are composed of two neighboring neurons: LGMD1 and LGMD2, both of which contain extensive dendrite trees with fan-like shapes within their pre-synaptic regions. There have been many computational models inspired by both of them, e.g., Rind and Bramwell (1996), Yue and Rind (2009), i Badia and Verschure (2004), Bermúdez i Badia et al. (2010), Fu and Yue (2015), and Fu et al. (2018).

Figure 1D shows a schematic of a typical LGMD1 model, illustrating the sequential process of signal transmission and processing. Initially, the input video signal passes through the photoreceptor (P) layer, symbolizing the perception of the luminance changes in each pixels. The signal then bifurcates into two distinct pathways within the inhibition/excitation (I/E) layer. This crucial junction serves to filter out irrelevant elements of movement, ensuring that only relevant information is passed on. The signals then converge in the summation (S) layer, signifying an activation process through which only the correct signals can pass. This layer acts as a critical node to reassemble and integrate the signals. Finally, the signals progress through the grouping (G) layer, culminating in the activation of the LGMD neuron. This model also contains an additional layer, the feed-forward inhibition (FFI), which shuts down the entire model if the visual stimuli becomes too large. Figures 1B, C shows its detection results against looming objects, where all looming objects are correctly detected in all results.

Similarly, a typical LGMD2 model is illustrated in Figure 1E. The LGMD2 neuron is the neighboring partner of the LGMD1, which is modeled as two pathways—the “on” and “off” pathway (O'Shea and Williams, 1974; O'Shea and Rowell, 1976). Compared to LGMD1, the signals of LGMD2 in the I/E and S layers are separated into on and off pathways then gathered in the S layer. This brings LGMD2 a unique characteristic that it only sensitives to dark objects approaching against a light background, and is not sensitive to white or light objects approaching against a dark background, representing a preference for light-to-dark luminance changes.

These models show complex intra-layer structures where each layer performs particular processing functions. For example, the LGMD1 model uses pattern convolution in its inhibition layers to imitate genuine inhibition processes observed in locust neurons. In this model, summation and averaging operations simulate the average reaction of similar locust neurons. These layers possess a close correlation with genuine neuronal response signals. Inter-layer connections within these models display consistent connectivity patterns such as one-to-one, one-to-many, and many-to-one connections. This can be Time delays of varying values are implemented for these connections to account for temporal aspects of information processing. Furthermore, global responses of insect neurons are depicted through cross-layer connections, acting as a switch to the models' output.

The models' functions are mainly linear, which we believe simplifies the computational procedures and supports the modeling of biological systems through linear transformations and responses. Both the LGMD1 and LGMD2 models, for instance, employ a summation process to depict the total response of each cell. Such a linear structure enables the development of a hyper-layer network representation in which the processing function of each layer includes input, processing and output. This integrated description permits a common expression of different properties found in different models, and enables model transformations by modifying these meta-properties. This framework highlights the potential of bio-plausible models to process information efficiently and adapt effectively, reflecting the computational proficiency of nature.

## 3 The strategic prototype: LGMD-UP

Based on the shared structures of the above-mentioned models, we believe that the construction of a unified description—The LGMD-Universally Prototype(LGMD-UP)—has become possible. To provide typical examples, we will employ the extensively examined LGMD1 and LGMD2 models.

The LGMD1 model typically comprises four network layers and a feed-forward global suppressor—the P, I/E, S, G layers, and the FFI suppressor. The input signal, post-processed in the P layer, passes to the I/E layer. At this stage, the signal splits into two pathways for specialized processing. The signals from these pathways merge in the S layer, culminating in a response in the G layer. At the same time, the FFI layer can be activated in response to excessive P-layer connections, effectively suppressing global signal output. The LGMD2 is similar, but it splits into two pathways in the I/E layer, then the signals are again gathered together in the S layer.

**Figure 2:**
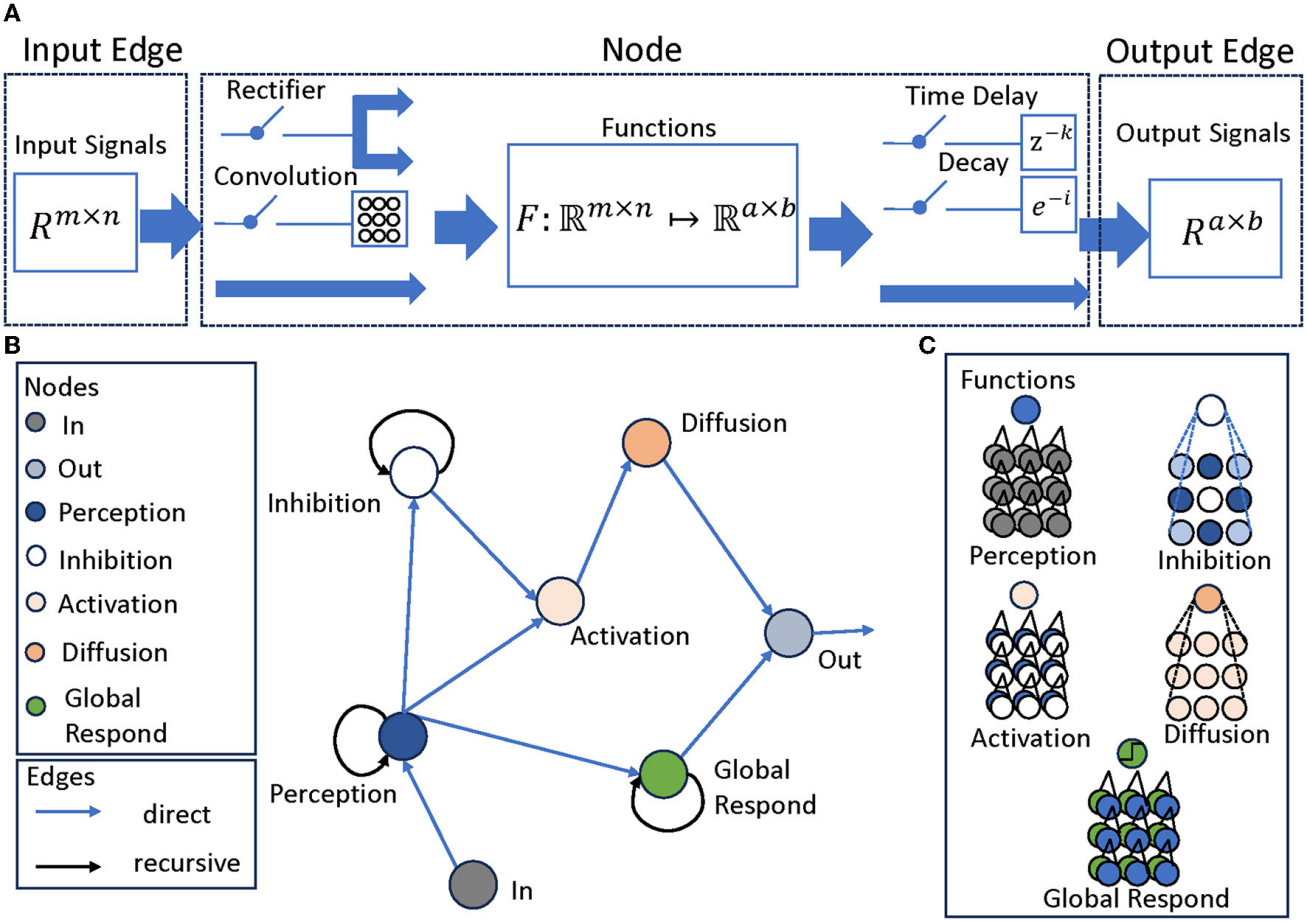
The structure of LGMD-UP. **(A)** Attributes of the Graph Network, including Input Edge, Node, and Output Edge, each attribute has different properties. Input Edge contain signals of the form *R*^*m*×*n*^, it is then go through the Node with a function *F*:ℜ^*m*×*n*^ ↦ ℜ^*a*×*b*^. There are two optional pre-processing parameters and two optional post-processing parameter. Output Edge are the signals of the form *R*^*a*×*b*^. **(B)** The LGMD models described in Graph Network. The Perception, Inhibition, and Global Respond Mechanisms are recursive structures, while other layers are feed-forward structures. **(C)** The functions in each node. They may differ from each other, but they all correspond to the layers of current LGMD models. For example, the Perception node calculates the differences between two inputs: its last output and the current in node.

To represent these structures coherently, we use a modular directed graph network (Scarselli et al., 2008), where each node corresponds to a layer, each with different inputs, processing methods and outputs. Nonetheless, a cohesive depiction can still be achieved through a modular representation, as presented in Figure 2A that acts as a summary framework for a solitary layer. This layer encompasses characteristics, for instance:

1. Input Edge attributes, specifying the source of the signals and the signal size.

2. Node attributes, defining the processing function. For each node, there are two optional pre-processing parameters, which is the convolution and the rectifier. There are also two optional post-processing parameters, which is the time delay coefficient and the decay coefficient.

3. Output Edge attributes, specifying the destination of the signal, and its size.

In each node, two optional pre-processing and two post-processing is applied. The pre-processing procedures, which are the convolution and the rectifier, can be formulated as follows:


$$f_{conv}(x)=x*K;f_{rectifier}(x)=\{\begin{array}{ll} max(0,-x), & if\;x\;⩽\;0;\\ max(0,x), & if\;x>0. \end{array}$$

where * is the convolution process, *K* is the convolution kernel with adjustable size. If *K*∈ℜ, then it's a gain coefficient.

These processes are purely optional, depending on the function and requirements of each node. For example, in LGMD2 models, rectifiers are enabled in the I/E and S layers and disabled in other layers. The convolution (including gain), on the other hand, is more commonly used in the P, I/E, G, and FFI layers in both LGMD1 and LGMD2 models.

Similarly, the post-processing procedures, including time delay and decay can be also formulated as follows:


$$f_{delay}\left(x\right)=x\cdot z^{-k};f_{decay}\left(x\right)=x\cdot e^{-i}$$

where *f*_*delay*_ are defined in Laplacian domain.

These processes are also optional, depending on each node's function and demands. For example, time delays are enabled in P and I/E layers in both LGMD1 and LGMD2, decays are used in P layers, FFI layers in both LGMD1 and LGMD2.

We can further formulate this graph network *G* by a standardized graph description: *G* = {*N, E*}, where *N* is the set of nodes, and *E* is the set of edges. Let *pr*_*w*_ represent the pre-processing function, *f*_*w*_ represent the processing function and *po*_*w*_ represent the post-processing function. Then, the hidden state *x* and the output *y* of node can be defined as follows:


$$x_{t}=pr_{w}\left(l_{t},x_{t-1}\right);y_{t}=po_{w}\left(f_{w}\left(l_{t},x_{t}\right)\right)$$

where *l*_*t*_ are the signals from its input edges, *y*_*t*_ is the output at time *t*. Note that *f*_*w*_ can be different in each node, *pr*_*w*_ and *po*_*w*_ are the combinations of optional functions, which are also different in each node.

Using this construction, we obtain a modular LGMDs representation that matches the responses of the original network, fully exposing all structures and connections, as shown in Figure 2B. The network now manifests as a graph network with four distinct meta-properties: Perception, Inhibition, Activation, and Diffusion, with an attached Global Response Mechanism, as shown in Figure 2C. Each mechanism is critical to the overall performance of the system, mirroring intricate processes observed in biological counterparts.

**Perception mechanism:** The Perception Mechanism represents the initial stage in the model, focusing on detecting luminance changes over time in each cell's domain. It calculates differences between successive frames to pinpoint areas where luminance changes, indicating motion.

**Inhibition mechanism:** This critical filter assesses candidates identified by the Perception Mechanism and excludes those that do not correspond to approaching movements. Essential for boosting the model's accuracy, it guarantees that only relevant motion cues are conveyed for further processing.

**Activation mechanism:** The activation mechanism categorizes and sorts the appropriate stimuli that have passed through the inhibition mechanism. It operates as a decision-making procedure by assessing the significance of the stimuli and recognizing ones that need additional processing.

**Diffusion mechanism:** The Diffusion Mechanism augments and arranges the outcomes from the Activation Mechanism. It enhances the selected stimuli and integrates them into a coherent representation of the approaching object. This mechanism is vital for delivering a thorough and precise depiction of the stimuli.

**Global respond mechanism:** The Global Respond Mechanism serves as a toggle switch for the entire network. It identifies scenarios where motion detection may be unnecessary or pose potential harm. In such cases, it temporarily deactivates the entire motion detection network, thus ensuring network focus and efficiency is maintained.

## 4 Perspective on the LGMD-UP

The modular design of the LGMD-UP forms the bedrock of its flexibility. With five modular nodes replacing the hyper-layered connections, it facilitates the independent development, testing, and integration of each module in the system, thereby enabling easy customization and scalability of the model. Additionally, the independence of the modules means that changes or improvements can be made in one area without disrupting the entire system. The modular structure of this system has significant advantages in research and development environments–it permits constant testing and iteration. The ability to modify and turn on/off individual parameters in each node ensures that the LGMD-UP can be tailored to meet specific requirements or easily adapt to new challenges.

The graph network framework significantly enhances the efficiency of the LGMD-UP when compared to traditional hyper-layered connections. Due to the independence of each node, this framework allows for easier time updates and parameter changes, which are essential in rapidly evolving fields such as swarm robotics. The graph network also simplifies and unifies structures, making it more efficient than more traditional designs. This efficiency is essential for keeping the visual system in line with technological advances and the diverse requirements of applications.

The adaptability of the LGMD-UP is also based on its graph-network structure, which allows the nodes and edges of the network to be dynamically reconfigured. This in turn establishes sophisticated interrelationships between the model's elements, optimizing processing for specific tasks or environments. For instance, within the context of automated driving, specific mechanisms, like the inhibition mechanism, can be adjusted and independently tested to match the current road and traffic conditions, without impacting other functions. Crucial to its adaptability, the ability to modify the network's configuration and connections according to a variety of demands optimizes the system's capabilities to adjust to various visual scenarios adeptly.

It is also worth noting the distinction between bio-inspired and bio-plausible models in this context. Bio-inspired models extract key biological principles for technological applications, whereas bio-plausible models aim for accuracy and plausibility in simulating actual biological processes. The LGMD model uniquely embodies both approaches, precisely imitates the LGMD neuron in insects that detects fast-approaching objects, thus demonstrating bio-plausibility. Simultaneously, it promotes technological progress in motion detection systems, thereby classifying it as a bio-inspired category as well. This duality highlights the special role of LGMD models in bridging biological accuracy and technological innovation.

## 5 Conclusion

In conclusion, our innovative approach to bio-plausible visual systems in robotics, which focuses on the LGMD models of Locusts, demonstrates significant potential for the advancement of robotic perception. The LGMD-UP, which incorporates these discoveries, represents a considerable advancement in our methodology for robotic vision systems. The graph network structure, which takes inspiration from nature's simplicity and efficiency, provides exceptional adaptability and modularity. This feature makes it highly suitable for addressing the dynamic demands of contemporary robotics. The LGMD-UP does not only improve robots' ability to interact with their environment in real-time but also paves the way for further research and development. Our view paves the way for further advancements in this field, encouraging continued exploration and refinement of bio-inspired systems for increasingly intuitive and adaptive robotic perception. Future work can be focused on this perspective, by re-framing and adjusting the LGMD-UP to meet specific requirements of diverse applications.

## Data availability statement

The original contributions presented in the study are included in the article/supplementary material, further inquiries can be directed to the corresponding author.

## Author contributions

HW: Data curation, Investigation, Software, Visualization, Writing—original draft. SY: Project administration, Writing—review & editing, Conceptualization, Funding acquisition, Resources. CH: Formal analysis, Funding acquisition, Resources, Validation, Writing—review & editing, Methodology.
